# Strain-transcending neutralization of malaria parasite by antibodies against *Plasmodium falciparum* enolase

**DOI:** 10.1186/s12936-018-2455-6

**Published:** 2018-08-20

**Authors:** Sneha Dutta, Aneesha Tewari, Chinthapalli Balaji, Reena Verma, Anasuya Moitra, Mamta Yadav, Prakhar Agrawal, Dinkar Sahal, Gotam K. Jarori

**Affiliations:** 1 Department of Biological Sciences, Tata Institute of Fundamental Research, Homi Bhabha Road, Colaba, Mumbai India; 2 International Center for Genetic Engineering and Biotechnology, Aruna Asif Ali Marg, New Delhi, India; 3 Present Address: Department of Genetics and Complex Diseases, Harvard T. H. Chan School of Public Health, Graduate School of Arts and Sciences, Harvard University, Boston, USA; 4 Present Address: Department of Biology, Whitehead Institute for Biomedical Research, Massachusetts Institute of Technology (MIT), Boston, USA

**Keywords:** *Plasmodium*, Enolase, Protective epitope, Monoclonal antibodies, Growth inhibition, Merozoites, Malaria vaccine

## Abstract

**Background:**

*Plasmodium* enolase is a target for the growth neutralizing antibodies. Interestingly, the three invasive stages i.e. sporozoites, merozoites, and ookinetes express this protein on their cell surface. Polyclonal anti-*Plasmodium falciparum* enolase (Pfeno) antibodies disrupt traversal of ookinete through mosquito mid-gut wall as well as have inhibitory effect on parasite growth at erythrocytic stage. In a recent study, it was observed that immunization with a unique epitope of parasite enolase (EWGWS) could confer partial protection against mouse malaria. Further validation is needed for the protective potential of this unique epitope in otherwise highly conserved enolase.

**Methods:**

In order to investigate the efficacy of growth inhibitory potential of the epitope of *P falciparum* enolase, a monoclonal antibody specific to EWGWS is generated. In vitro parasite growth inhibition assays and passive immunization of *Plasmodium yoelii* (or *Plasmodium berghei*) infected mice were used to assess the parasite growth neutralizing activity of the antibody.

**Results:**

Screening a panel of monoclonal antibodies raised against recombinant Pfeno that were specific to EWGWS resulted in isolation of H12E1. This antibody recognized only EWGWS epitope containing enolases. H12E1 strongly inhibited parasite growth in culture. This inhibition was strain transcending. Passive infusion of this antibody in *P. yoelii* or *P. berghei* infected mice showed significant reduction in parasitemia as compared to controls (p < 0.001). Surface Plasmon Resonance measurements indicated high affinity binding of H12E1 to *P. falciparum* enolase (K_D_ ~ 7.6 × 10^−9^M).

**Conclusions:**

A monoclonal antibody directed against EWGWS epitope of Pfeno was shown to inhibit the growth of blood stage malarial parasites. This inhibition was species/strain transcending and is likely to arise due to blockade of enolase on the surface of merozoites, functionally implicating Pfeno in invasion related events. Presence of enolase on the cell surface of merozoites and ookinetes could potentially result in inhibition of host cell invasions at erythrocytic and transmission stages in the parasite life cycle. It is suggested that antibodies against EWGWS epitope have the potential to confer dual stage, species and strain transcending protection against malaria.

**Electronic supplementary material:**

The online version of this article (10.1186/s12936-018-2455-6) contains supplementary material, which is available to authorized users.

## Background

Despite recent progress in malaria prevention and control, the disease continues to take a heavy toll [1]. It is believed that the development of a malaria vaccine, which is effective over a wide range of human populations and covers a vast genetic diversity of the parasite, would be essential for complete eradication of malaria. At present, the best available vaccine is RTS,S/AS01, which received a positive opinion from European regulators for the first time in 2015 [2], is a pre-erythrocytic vaccine that targets sporozoites and protects by curtailing liver infection [3]. In recent field trials, this vaccine had shown modest efficacy in protection [4–6] and is unlikely to meet the goals for complete eradication of malaria. Efforts to develop a vaccine against the asexual blood stages of the parasite (which causes the clinical symptoms of the disease and against which natural immunity evolves) have led to identification of several antigens that could induce protective response. Some of these have been tested for their protective activity without much success [7–12]. Two major hurdles in the path for the development of a blood stage vaccine have been the presence of a high degree of antigenic polymorphism in the parasite and the high threshold levels of antibodies needed for protection [13, 14]. *Plasmodium,* being an intracellular parasite, needs to invade host cells to establish infection. There are three invasive stages (sporozoites, merozoites, ookinetes) in the life cycle of *Plasmodium*, two of which (sporozoites and merozoites) get briefly exposed to the humoral branch of the human immune system, rendering the molecular machinery of merozoites and sporozoites involved in invasion as attractive targets for anti-malarial vaccine.

Current approaches to circumvent the barriers imposed by the genetic diversity in *Plasmodium* and its multistage complex life cycle are to combine multiple antigens that are valid targets at various stages in the parasite life cycle as well as their orthologues from different species/strains to obtain an effective multistage, species and strain transcending malaria vaccine [15–17]. An alternative approach will be to identify antigens or epitopes that have cell surface expression at multiple stages, do not exhibit polymorphism, have critical non-redundant physiological function(s) and have high immunogenicity.

Pfeno has recently been identified to be a target of parasite neutralizing antibodies. This antigen is unusual in exhibiting cell surface expression at all the three invasive stages [18–20]. Structurally, Pfeno is distinct from the host (human and mosquito) enolases in having a plant-like insert, EWGWS [21, 22]. Enolase in merozoites and ookinetes is a target for parasite neutralizing antibodies [19, 20, 23]. Anti-rPfeno antibodies showed strong growth inhibitory effect on blood stage in vitro cultures of *Plasmodium falciparum*. Antibodies induced by active immunization with rPfeno resulted in significantly reduced parasitemia that prolonged the survival of the infected mice [20]. These mice had a large fraction of their IgGs targeted against the EWGWS epitope of parasite enolase [24]. Immunization with a peptide containing EWGWS epitope also resulted in control of parasitemia. Antibody titers in EWGWS immunized animals showed a positive correlation with survival and a negative correlation with parasitemia [23]. Partial protection observed in immunized mice suggested that either the induced antibodies were at sub-threshold levels (poor immunogenicity) or there was a functional redundancy in the invasion step at which enolase was involved. Here, a monoclonal antibody based approach is taken to obtain further insight and assess the merozoite invasion neutralizing potential of the antibody directed against EWGWS epitope.

## Methods

### Materials

Protein A-Sepharose was obtained from Thermo Scientific, Rockford, IL, USA. ABTS was supplied by Sigma-Aldrich, St. Louis, MO, USA. Synthetic peptides were supplied by USV India Ltd., Mumbai, India. All the chemicals used were of analytical grade. *Halobacterium* gas vesicle nanoparticles (wild type and recombinant) were prepared as described earlier [23]. Recombinant particles had a peptide with a sequence ASKN**EWGWS**KSKS cloned in one of the gas vesicle proteins (gvpC).

### Purification of rPfeno and activity measurements

rPfeno was purified using the over expression system as described earlier [21]. Briefly, full-length enolase gene was cloned from a gametocytic cDNA library made from NF54 strain of *P. falciparum*. The cloned  gene was over expressed in *E. coli* BL21 and the 6XHis tagged recombinant protein was purified using Ni–NTA metal affinity chromatography. Cloning resulted in addition of 18 aminoacid residues (MRGSHHHHHHGSACELGT-) to the N-terminus and 7 residues (-LQPSLIS) to the C-terminus. Enolase activity was measured using 2-phosphoglycerate (2-PGA) as the substrate and monitoring the formation of phosphoenolpyruvate by increase in OD at 240 nm. The assay mixture consisted of 500 μl of 50 mM Tris–HCl pH 7.5, 1.5 mM Mg(II) and 1.5 mM 2-PGA [24].

### Generation of mouse mAbs against rPfeno

For the generation of mAbs directed against various epitopes of rPfeno, 6 weeks old, female BALB/c mice were injected intraperitoneally with 50 µg of purified rPfeno emulsified with Freund’s complete adjuvant. This was followed by booster injections at an interval of 3 weeks for the next 2 months. The best responder mouse was immunized with 250 μg of immunogen (rPfeno) in phosphate buffer saline (PBS) (10 mM Na-Phosphate, 137 mM NaCl, 2.7 mM KCl, pH 7.4). Five days later, the splenocytes from this mouse were fused with mouse myeloma SP2/O-Ag14 cells (Sigma-Aldrich) using polyethylene glycol (Merck) as fusogen. After selection in medium containing Hypoxanthine-Aminopterin-Thymidine (HAT, Invitrogen) for a week, the resulting hybrid clones were screened for antibody secretion wherein binding of hybridoma cell culture supernatants to rPfeno was tested by ELISA. Of the fifty seven hybrid clones that showed reactivity to rPfeno in ELISA, thirty four were re-cloned by limiting dilution to obtain pure clones. Generation of hybridomas and production of hybridoma supernatants were carried out by Bioklone Biotech Pvt. Ltd, Chennai, India.

### Purification and isotyping of mAbs

mAbs from the hybridoma supernatants were purified using the ammonium sulfate precipitation method [25]. Briefly, the IgG (mAb) was precipitated from hybridoma supernatant by adding powdered ammonium sulfate to 45% saturation using standard protocol. The precipitate was collected by centrifugation, dissolved in phosphate saline buffer (PBS) and dialyzed to remove the salt. The dialyzed solution was passed through a Protein-A affinity column (Thermo Scientific Pierce 20356) and eluted with a low pH buffer (0.1 M glycine–HCl, pH 2.0). Alternatively, the ascites fluid was filtered through glass wool and ~ 15 ml filtrate was passed through 5 ml Protein-A Sepharose column. The flow through was collected and stored for later testing of any unbound residual antibody. The column was washed with three column volumes of PBS. Following this, 5 ml of elution solution (0.1 M glycine–HCl pH 2.0) was added and 1 ml fractions of the eluate were collected. Each 1 ml fraction was neutralized with 100 μl of 1 M Tris, pH 9.0. 10 μl of each of the five aliquots, dissolved in Laemmli buffer [26], were run on a 10% SDS-PAGE to evaluate the purity of the preparation. IgGs were also purified from anti-rPfeno antisera (polyclonal) as well as pre-immune sera using ammonium sulfate precipitation and Protein A affinity chromatography as described above. Purified IgGs were sterilized by filtration through a 0.22-μm filter and the solution was tested in LB medium for any bacterial contamination.

For determining the IgG subtype of mAb, 100 μl of goat antibodies to mouse isotypes viz., IgG1, IgG2a, IgG2b, IgG3, IgM and IgA were coated in the wells of a microtitre plate at 1:1000 dilution. Following this, the hybridoma culture fluid containing anti-rPfeno mAb was added and incubated overnight at 4 °C. Mouse anti-rPfeno serum (1:1000) was used as positive control. The wells were later treated with rabbit anti-mouse IgG-HRP (that binds to mouse IgG, IgM and IgA) and ABTS substrate was added to measure reactivity at 405 nm [21]. The OD values obtained for the positive control were IgG1: 0.61; IgG2b:0.69; IgG3:0.57; IgM:2.24; IgA:0.93 indicating the ability of secondary antibody to bind to all three immunoglobulins. Isotyping of mAbs was performed by Bioklone Biotech Pvt. Ltd, Chennai, India.

### ELISA

ELISA was performed as described earlier [20]. Briefly, wells in immunoplates (Nunc, Denmark) were coated with 50–100 ng of purified rPfeno and incubated at 37 °C for 2 h. After a single wash, the wells were blocked by the addition of 5% skimmed milk in PBST (phosphate buffer saline containing 0.05% Tween-20, pH 7.4) and incubated for 1 h at room temperature or 4 °C overnight. The blocking solution was discarded and the wells were washed with PBST 3 times for 5 min each. The wells were then coated with 100 μl of primary antibody (dilution varied according to experiment) and incubated at room temperature for 1 h. The solution was discarded and the wells were washed with PBST three times to remove any unbound antibody. This was followed by coating the wells with HRP conjugated mouse secondary antibody at a dilution of 1:1000 and incubating it at room temperature for 45 min. The solution was discarded and the wells were washed with PBST thrice; 200 μl of ready-to-use ABTS substrate was added into each well. The colour was allowed to develop for 10–15 min and the OD was measured at 405 nm on a Tecan Plate Reader.

For a competitive ELISA, a cyclized 13-mer peptide with a sequence NH_2_-SCKN**EWGWS**KSCS-COOH (CPS1920) containing EWGWS was synthesized. ELISA plates were coated with 50 ng of rPfeno (1 pmol); 100 µl of buffer containing 1 pmol of antibody and different concentrations of CPS1920 peptide (0, 5, 10 and 100 µM) were incubated overnight at 20 °C. Pre-incubated peptide plus mAb was then added to the ELISA plate wells in triplicates and incubated for 10 min and the plates were developed using the ABTS substrate as described above. An irrelevant peptide NH_2_-SWPLPSHTAVWG-COOH (Peptide CPS1916-1) was used in place of CPS1920 as a control to ensure specificity of competitive displacement.

### SDS-PAGE and western blotting

Proteins were resolved on a 10 or 12% SDS-PAGE [26] and visualized by staining with Coomassie Brilliant Blue R-250. For Western blotting, proteins separated by SDS-PAGE were transferred to a PVDF membrane using semi-dry western transfer apparatus (Trans-blot SD-cell, Bio-Rad Laboratories, Inc., Hercules, CA, USA) at a constant voltage of 18 V for 50 min. The membranes were blocked with 5% skimmed milk in PBST for 2 h. The blots were treated with primary antibody followed by washing and incubation with HRP conjugated secondary antibody. The immunoblots were developed using ECL substrate (Pierce).

### In vitro culture and synchronization of *Plasmodium falciparum* 3D7

*Plasmodium falciparum* 3D7 was cultured as described earlier [27]. Fresh human RBCs were obtained from the blood samples drawn from consenting informed volunteers by a professional at the Pathology Department, Health Promotion Facility of Tata Institute of Fundamental Research, Mumbai, India.

The ring stage parasite cultures were synchronized by sorbitol treatment as described earlier [28]. Briefly, 5% sorbitol was added to the infected RBC pellet and incubated at 37 °C for 10 min followed by centrifugation (1500 g, 5 min). The cells in the pellet were cultured for 48 h (i.e., one cycle of multiplication) before subjecting them to a second round of sorbitol treatment. Such a double sorbitol treatment resulted in a high degree of synchrony (> 98% ring stage parasites) [29].

### Growth inhibition assays

Asexual stages of *P. falciparum* 3D7 were cultured in vitro [30] and growth/invasion inhibition assay was performed on a 96-well plate as described earlier [16, 20, 31]. *Plasmodium falciparum* 3D7 cultures maintained on O^+^ erythrocytes at 2% haematocrit were synchronized at the ring stage using sorbitol treatment [29]. For each assay, 200 μl of 0.5–3% parasitized cells were used in three identical wells for each time point or antibody concentration. The culture plates were incubated for 48 h and parasitemia was assessed by examining the culture smears using Field stain [32]. Percent parasitemia was determined by counting at least > 1000 erythrocytes [20]. Differences in mean parasitemia between control (no mAb) and experimental (mAb added) samples were analysed with Student’s t-test using GraphPad (InStat, San Diego, CA, USA). Differences were considered significant if the p-values were < 0.05.

For the data presented in Fig. 4, the following method was used to measure the GIA of the mAb. Chloroquine (CQ) sensitive (3D7) and resistant (INDO) strains of *P. falciparum* were used for in vitro cultures. Parasite strains were cultivated by the method of Trager and Jensen [33] with minor modifications. Cultures were maintained in fresh O^+^ human erythrocytes (obtained from Rotary Blood Bank, 56-57, Tughlakabad Institutional Area, New Delhi, 110062). Cultures were maintained at 4% haematocrit in complete medium (RPMI 1640 with 0.2% sodium bicarbonate, 0.5% Albumax II, 45 mg/l hypoxanthine, and 50 mg/L gentamicin) at 37 °C under reduced O_2_ (gas mixture 5% O_2_, 5% CO_2_, and 90% N_2_). Stock solutions (1 mM) of CQ were prepared in water (MilliQ grade), and lyophilized powders of antibodies were dissolved in filter sterilized MilliQ water to a final concentration of 1 mg/ml. 0–6 µl antibody solutions were transferred to wells of microtitre plate taking each dose in triplicates and final volume was made to 6 µl in each well using PBS. Chloroquine (1 µM) was used as positive control. Ring stage Sorbitol synchronized culture (94 µl) was aliquoted to wells of 96-well plate at 2% haematocrit and 1% parasitemia in a final volume of 100 µl. After 48 h of incubation under standard culture conditions, plates were harvested and read by the SYBR Green I fluorescence-based method [34, 35] using a 96-well fluorescence plate reader (Victor, Perkin-Elmer), with excitation and emission wavelengths of 485 and 535 nm, respectively. The fluorescence readings were plotted against antibody concentration and EC_50_ values were obtained by visual matching the mAb concentration corresponding to 50% growth inhibition. In cases where parasitemia was determined microscopically, > 2000 cells were counted using Auto count software [36].

### Passive immunization

Passive transfer of immunity was tested using 6–8 weeks old Swiss male mice. In two different experiments, mice were challenged either with *P. yoelii* 17XL or with *P. berghei* ANKA. Frozen stocks of parasite infected RBCs (pRBCs) were thawed and injected into the mice. The pRBCs obtained from the second round of passage were used to challenge the mice. In the first experiment (Fig. 5a), all mice (five animals in experimental group, three each in pre-immune and no IgG control groups) were administered with ~ 10^5^ *P. yoelii* 17XL infected pRBCs. On the same day, purified antibody (0.75 mg H12E1 in 100 µl) was administered intravenously. Control groups were injected either with 0.75 mg of pre-immune IgGs or with 100 µl of buffer.

In the second experiment (Fig. 5b), two groups of mice (five animals in experimental group and five in control group) were challenged with ~ 10^5^ *P. berghei* ANKA infected mouse pRBCs. H12E1 antibody (1 mg in 100 µl) was injected in experimental group of animals while the control group received 1 mg of purified pre-immune IgGs. In both the experiments, thin blood smears were prepared (by obtaining blood via the tail bleeding method) every alternate day and the parasitemia was measured using *Plasmodium* Auto count software [36]. At each time point > 2000 cells were counted. Percentage parasitemia was determined as an average of three samples and plotted with standard deviation at each time point. Differences in mean parasitemia between the control and experimental groups were analysed with Student’s t-test as described above. If parasitemia rose to ≥ 50–60% and the mouse showed severe clinical symptoms, the animal was euthanized.

### mAb-rPfeno interaction using Surface Plasmon Resonance

Surface Plasmon Resonance (SPR) measurements were made using Biacore T20 machine version 2 control and evaluation software (GE Healthcare Life Sciences, Sweden) located at IIT, Mumbai. All measurements were made at 25 °C. Running buffer consisted of 10 mM HEPES containing 150 mM NaCl, 3 mM EDTA and 0.005% (w/v) P20 surfactant, pH 7.4. rPfeno was immobilized covalently on the surface of CM5 sensor chip using amine coupling with the target response unit of 1000 Rmax. The process involving steps of activation, immobilization and blocking were carried out. Briefly, using a flow rate of 10 μl/min, the chip surface was activated by injecting freshly prepared 1:1 mixture of EDC and NHS (both dissolved in water as per the manufacturer’s instructions). Subsequently, 30 μg/ml rPfeno in 10 mM sodium acetate (pH 5.5) was passed through the active flow cell for 90 s at a flow rate of 30 μl/min. The remaining activated carboxy methyl groups on the surface were blocked by a 7-min injection of 1M-ethanolamine-HCl, pH 8.5. An unmodified flow cell surface was used as a reference for each analysis to check for the non-specific binding response to dextran matrix.

For the measurement of kinetics of interaction between rPfeno and mAb, the analyte (mAb H12E1) was diluted with HEPES buffer and injected over the rPfeno-immobilized chip for 90 s at a flow rate of 30 µl/min followed by a final 240 s dissociation phase. Several concentrations of analyte ranging between 0.78 and 100 nM were used. Regeneration was done with 10 mM NaOH at a flow rate of 30 µl/min for 30 s. Data were evaluated with BIA evaluation software (GE Healthcare Life Sciences, Sweden version 2.0). The sensograms obtained at each antibody concentration was fitted with 1:1 bivalent kinetics for both the association and dissociation phases. Equilibrium dissociation constant (K_D_) was then calculated from the dissociation and association rate constants.

### Molecular modelling

3D-structure of Pfeno was modelled based on the X-ray crystallographic structure of *T. gondii* Enolase1 (TgENO1; PDB: 3OTR) [37] using the Automated Mode of Swiss Model PDB viewer 8.05 (http://swissmodel.expasy.org/). UniProt/Swiss-Prot database was used to obtain the amino acid sequence of Pfeno (UniProt Accession No. Q27727, 446 amino acids). Energy minimization of the optimized model structure was achieved using the software: MOE 2016.08 (Chemical Computing Group, Canada). PyMOL 1.3 [38] was used to visualize the modelled structures.

## Results

### Screening of hybridoma supernatants for EWGWS specific mAbs

**Fig. 1:**
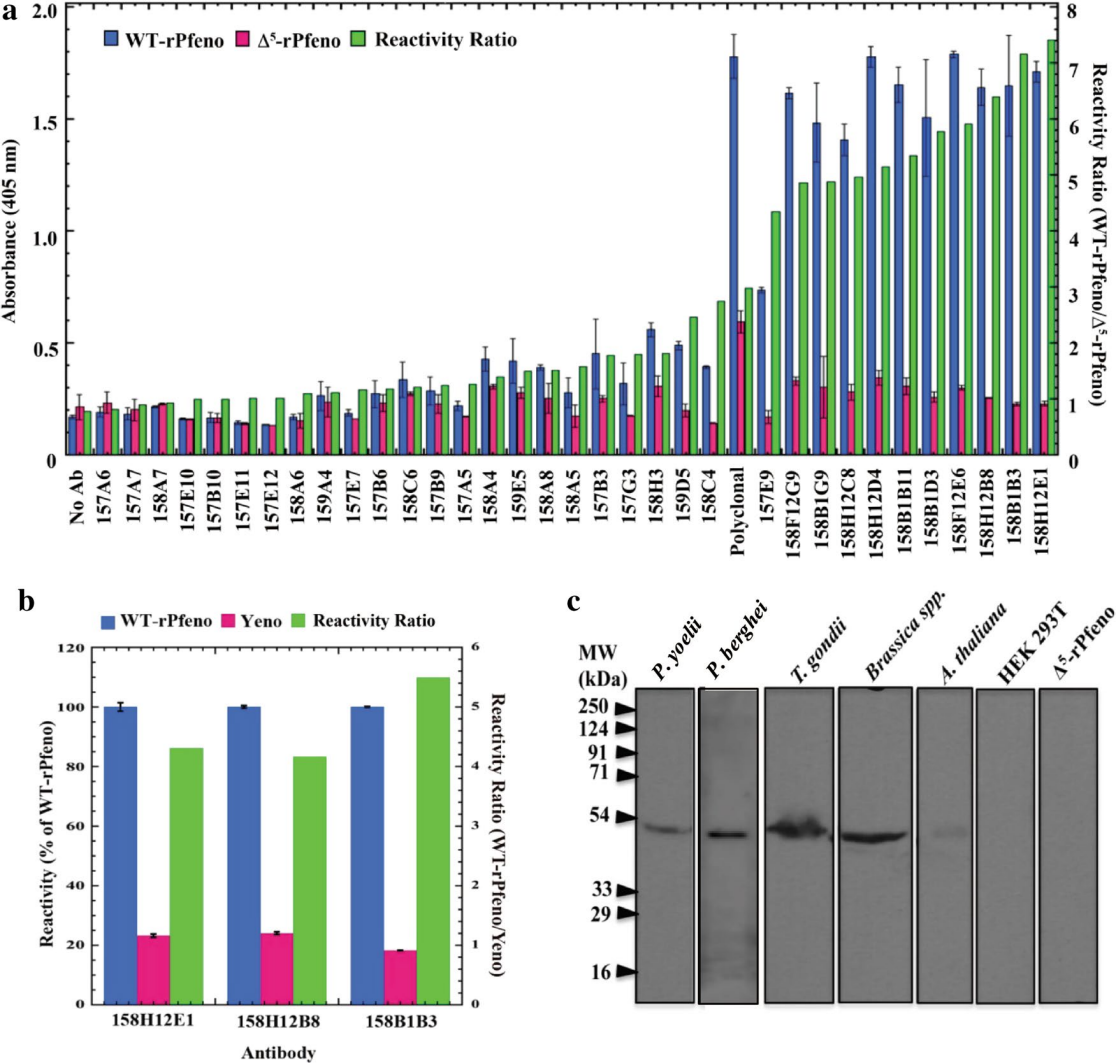
Screening of mAbs that are specific to the EWGWS epitope in Pfeno. **a** Reactivity of mAbs with WT-rPfeno and Δ^5^-rPfeno as measured by ELISA. Reactivity ratio (green bars) is defined as the ratio of the two reactivities, i.e., O.D._WT-rPfeno_/O.D. _Δ5-rPfeno_. **b** Reactivity of anti-rPfeno monoclonal antibodies with yeast enolase that lacks the insert sequence. Three mAbs with high reactivity ratio namely H12E1, H12B8 and B1B3 were used in this ELISA. **c** Western blot analysis of cell lysates from different organisms (*P. yoelii, P. berghei, Toxoplasma gondii, Brassica* spp., *Arabidopsis thaliana*, Human Embryonic Kidney cells (HEK 293T) and ∆^5^-rPfeno). Blot was probed using anti-rPfeno mAb H12E1. Antibody reactivity is observed only for the enolases containing the EWGWS motif. Enolases lacking this insert (HEK 293T and Δ^5^-rPfeno) did not show any reactivity

For the identification of mAbs that were directed against the EWGWS epitope in Pfeno, supernatants from 34 Pfeno-positive hybridoma clones were tested for their reactivity with WT-rPfeno and ∆^5^-rPfeno (a deletion variant lacking EWGWS) [24]. The difference in reactivity of mAbs between rPfeno and ∆^5^-rPfeno presumably reflected the selectivity towards the insert sequence. Ratio of the reactivities with the two forms (rPfeno/∆^5^-rPfeno) was computed to identify the ones that were selective towards EWGWS. Figure 1a shows data for all hybridoma supernatants along with their reactivity ratios (rPfeno/∆^5^-rPfeno). There were three hybridoma supernatants that had ~ six to seven-fold greater sensitivity towards WT-rPfeno compared to the deletion variant ∆^5^-rPfeno and were of high binding specificity to EWGWS. These mAbs, i.e., H12E1, H12B8 and B1B3 that had reactivity ratios of 7.4, 7.2 and 6.4, respectively, were all of the IgG2b type (Additional file 1).

As deletion of EWGWS dissociates dimeric rPfeno into monomers, there is a possibility that the observed low reactivity could arise from altered epitope structure of ∆^5^-rPfeno [24]. To rule out such a possibility, the reactivity of EWGWS-specific mAbs against another dimeric enolase that does not have the EWGWS insert sequence, namely yeast enolase (Yeno) was measured. Parallel ELISA measurements were made using rPfeno and Yeno as antigens. As shown in Fig. 1b, all three antibodies showed high reactivity towards rPfeno compared to Yeno. EWGWS containing rPfeno had ~ 4.2–5.5-fold greater reactivity than Yeno that lacked the insert. In the initial experiments, all three mAbs were tested for their binding specificity and ability to inhibit parasite growth in culture. Since, most reproducible results in multiple such assays were obtained with H12E1, all subsequent experiments were performed using H12E1 antibody only.

### Western analysis of whole cell extracts using H12E1 antibody

To assess whether the H12E1 antibody exclusively recognizes only the EWGWS containing enolases, whole cell extracts of *Plasmodium yoelii, Plasmodium berghei, Toxoplasma gondii, Brassica* spp., *Arabidopsis thaliana* and human embryonic kidney cells (HEK293T) were subjected to western analysis using H12E1 mAb. The deletion variant ∆^5^-rPfeno was also included as a negative control lacking the EWGWS insert. The resulting blot showed a positive signal at ~ 50 kDa in, *P. yoelii, P. berghei, Toxoplasma gondii*, *Arabidopsis thaliana* and *Brassica* spp. (Figure 1c). Lanes containing HEK 293T cell extract and purified ∆^5^-rPfeno had no H12E1 reacting band. There were no other cross-reacting proteins in any of the cellular extracts indicating exclusive reactivity of the H12E1 to EWGWS epitope in enolases (Fig. 1c).

### Effect of insert sequence variation on binding of mAb with rPfeno

**Fig. 2:**
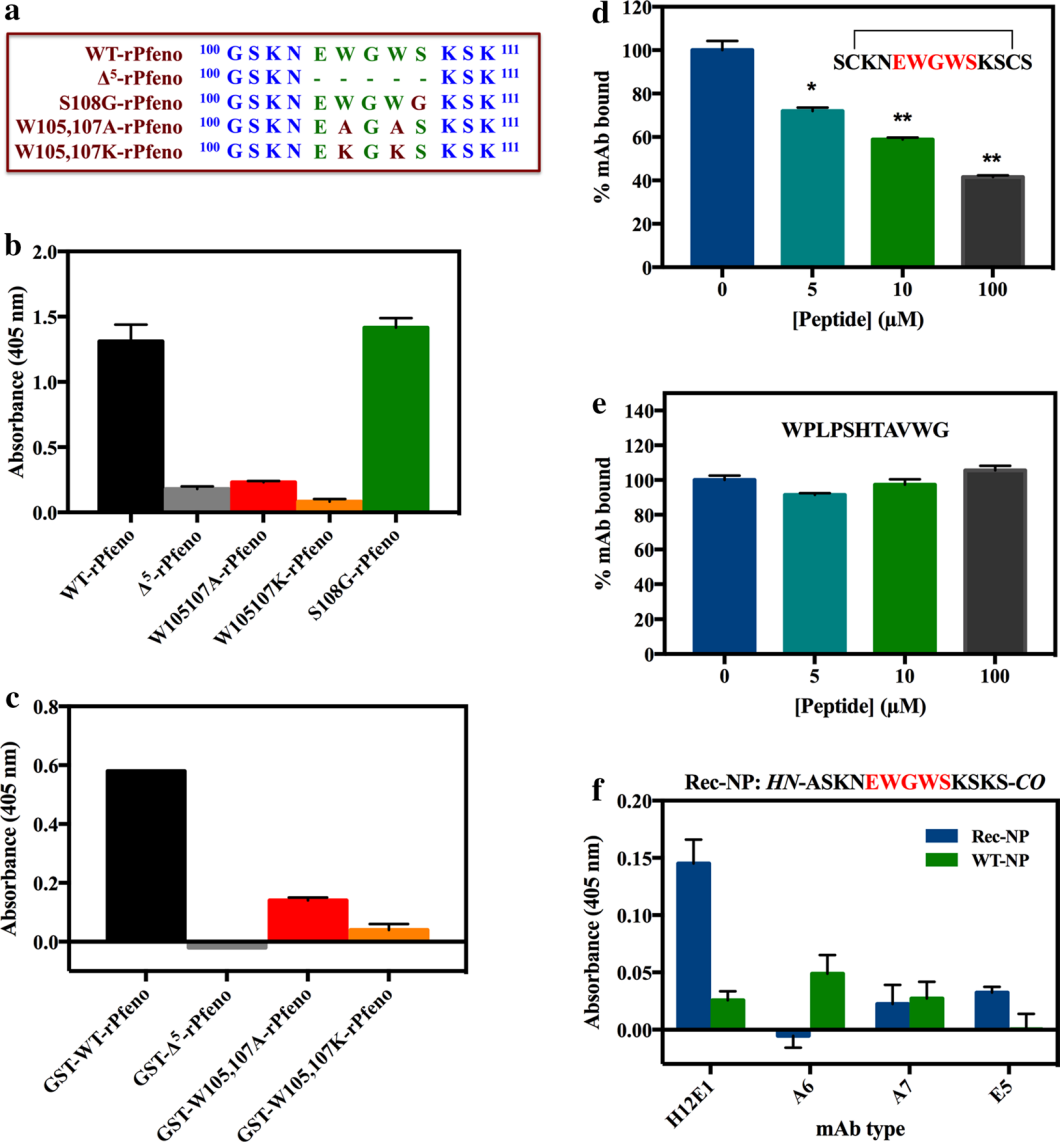
Antigenic specificity of mAb H12E1. **a** Sequences of pentapeptide insert in different variants. **b** ELISA of mAb binding to different variants of rPfeno. Oligomeric state of WT and S108G-rPfeno was dimeric while ∆^5^-rPfeno, W105, 107K-rPfeno and W105, 107A-rPfeno were in the monomeric form. 15 ng of purified protein of each variant form was coated on the plates. **c** ELISA of mAb binding to GST-tagged forms of rPfeno variants. In GST-tagged forms, all variants of rPfeno formed dimers (or higher oligomers). Wells were coated with 5 ng of purified protein of each variant. Variation in insert sequence affected the mAb binding while oligomeric state (monomer or dimer Pfeno) did not have any effect on reactivity. **d** Competitive ELISA with EWGWS containing peptide led to displacement of H12E1 from rPfeno. Peptide concentration was varied from 0-100 µM. **e** Titration with an irrelevant peptide epitope resulted in no displacement of the mAb by the irrelevant peptide. **f** Reactivity of various mAbs with WT-NPs and Rec-NPs. mAb directed against EWGWS i.e. H12E1 showed strong reactivity to the EWGWS containing Rec-NPs (incorporated with a clone peptide sequence ASKNEWGWSKSKS) as compared to WT-NPs

The effect of varying the insert sequence in Pfeno on binding of H12E1 was examined by monitoring the extent of antibody reactivity with different variants. Figure 2a shows the sequence changes made in these variants using site directed mutagenesis [21, 24, 39, 40]. Replacement of S108 with glycine (S108G-rPfeno) had no effect on antibody binding. However, altering the two tryptophan residues (W105 and W107) significantly reduced the binding ability of the variants with the mAb (Fig. 2b). These variants differ from each other in their oligomeric structure as well as in enzymatic activity. WT and S108G-rPfeno are enzymatically active and dimeric while the other three variants are monomeric and largely inactive [24, 39, 40]. To rule out the possibility that non-reactivity of the ∆^5^-rPfeno and Trp variants (W105, 107A-rPfeno and W105, 107K-rPfeno) was due to the altered oligomeric structure of the protein, an ELISA was performed on the GST-tagged forms of the different variants. In the GST tagged form, all these variants form dimers (or oligomers) and acquire active conformation [24, 39]. ELISA data presented in Fig. 2c indicated that stabilization of ∆^5^-rPfeno and the Trp variants as dimers did not improve their ability to bind to the antibody. Loss of H12E1 binding to rPfeno variants with altered insert sequence suggests that this antibody is specific for EWGWS.

### Competitive displacement of rPfeno by EWGWS-containing peptide

In a competitive ELISA experiment, the ability of an EWGWS sequence containing peptide NH_2_-SCKN**EWGWS**KSCS-COOH (cyclized by a disulfide bond) to compete with rPfeno, for binding to H12E1 was examined. With increasing concentrations of the peptide, there was a gradual decrease in the amount of H12E1-rPfeno complex formed. At the highest concentration of the peptide used, a decrease of ~ 45–50% in the binding of H12E1 was observed (Fig. 2d). Similar titration using an irrelevant peptide NH_2_-SWPLPSHTAVWG-COOH failed to compete with rPfeno for binding to the mAb (Fig. 2e). The observed competitive reduction in the amount of the bound mAb to rPfeno with increasing concentration of the peptide reflects the specific binding of mAb to EWGWS.

### Recognition of EWGWS by mAb H12E1

Further evidence for the binding specificity of H12E1 with EWGWS was obtained by measuring the reactivity of the antibody to the wild type (WT-NP) and recombinant nanoparticles (Rec-NP) [23]. These particles that were derived from *Halobacterium* gas vesicles had a peptide sequence ASKN**EWGWS**KSKS containing EWGWS cloned in one of its component proteins gvpC (Rec-NP). In an experiment where ELISA plates were coated with WT-NPs and Rec-NPs, reactivity of H12E1 and three other mAbs (A6, A7 and E5) that did not show any selectivity between wild type rPfeno and ∆^5^-rPfeno, was measured. ELISA values obtained with WT-NP and Rec-NP are shown in Fig. 2f. H12E1 showed ~ 7–8 times higher reactivity with Rec-NPs as compared to WT-NPs. Three other mAbs that were not specific for-EWGWS-did not show any reactivity with the two classes of the nanoparticles.

H12E1 mAb’s ability to discriminate between rPfeno and ∆^5^-rPfeno as well as the positive recognition of the peptides containing EWGWS were indicative of its high binding specificity to the unique epitope (EWGWS) of the parasite enolase.

### Growth inhibitory activity (GIA) of anti-enolase antibodies

**Fig. 3:**
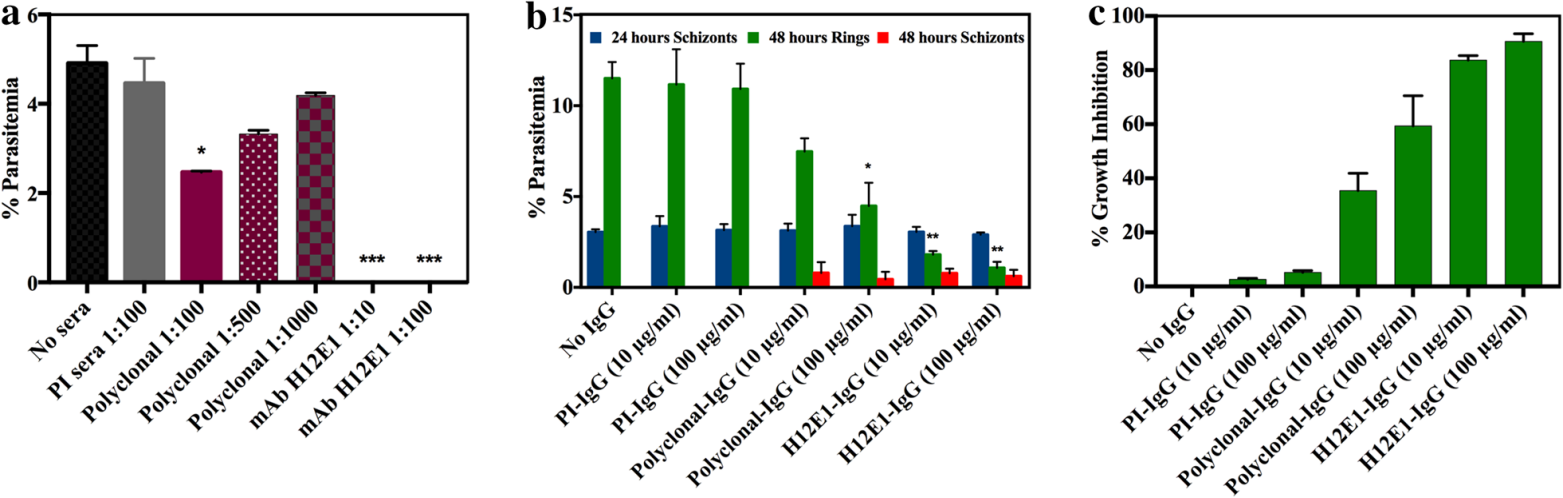
Growth inhibitory activity (GIA) of anti-Pfeno antibodies. **a** Effect of polyclonal anti-rPfeno sera and mAb H12E1 hybridoma supernatant on in vitro growth of *P. falciparum* 3D7. ‘Pre-immune (PI) serum’ and ‘no serum’ samples served as controls. Cultures were allowed to grow for 48 h before determining the % parasitemia. Initial parasitemia was 0.5%. **b** Effect of purified IgGs on parasite growth. Initial parasitemia was 3.2%. % parasitemia was determined at 24 (blue bars) and 48 h (green and red bars). At the 24-h time point all the samples had only schizonts. At 48 h, no IgG and pre-immune IgGs treated samples (contols) showed only ring stage parasites (green bars). Samples treated with polyclonal anti-rPfeno and H12E1 IgGs had rings (green bars) as well as schizonts (red bars) in parasitized cells. **c** % growth inhibition by different IgGs. Significance of decrease in parasite growth in antibody treated samples was compared with control samples using Student’s t-test. *p < 0.05, **p < 0.01 and ***p < 0.001

Effect of various antibodies on in vitro growth was measured in a growth/invasion inhibition assay. Addition of polyclonal anti-rPfeno antibodies and hybridoma supernatant from H12E1 at different dilutions on parasite growth is shown in Fig. 3a. Results obtained here were consistent with an earlier report [20] where ~ 50% inhibition in parasitemia was observed for the polyclonal anti-rPfeno antibody at 1:100 dilution. The observed inhibitory effect of H12E1 supernatant (1:10 and 1:100) treated samples was very potent. It showed almost complete inhibition of growth (Fig. 3a).

The possibility of such strong inhibition arising due to the presence of some toxicant in the hybridoma supernatant was considered. To rule out such a possibility, IgGs were purified and filtered through 0.22 µm filter to eliminate all components of hybridoma culture medium. The antibody solution was tested in LB medium to rule out the possibility of any bacterial contamination. Another experiment was performed using purified IgGs. In this experiment initial parasitemia was kept at ~ 3% and culture smears were prepared at 24 and 48 h post antibody addition, respectively. In the intra-erythrocytic asexual multiplication cycle, the parasite develops into a schizont from the mature ring stage in ~ 24 h. By 48 h, mature schizonts would have released merozoites that would re-invade fresh red blood cells (RBCs) and form ring stage *Plasmodia* in infected RBCs. In smears prepared at the 24-h time point all samples showed only schizonts (blue bars in Fig. 3b). There were no ring stage parasites in any of the samples indicating that the ring to schizont stage transition was not affected by the treatment with polyclonal or monoclonal anti-rPfeno antibodies. At 48 h the smears of both the control samples (no serum and pre immune serum) showed abundance of ring stage parasites (~ 12%) without any schizonts. Enolase antibody treated samples had relatively fewer numbers of parasites indicating the inhibition of growth. Interestingly, these smears also had some un-ruptured schizonts (~ 15–30% of total schizont population at 24 h) (Additional file 2). In case of H12E1 treated samples (IgG ~ 10 and 100 µg/ml), culture smears had very few rings and some mature unruptured schizonts (~ 15–30% of total schizont population at 24 h) (Additional file 2). Quantitative data of ring and schizont stage parasites at 24 and 48 h for all the samples are shown in Fig. 3b. Data are also plotted as % growth inhibition taking control parasite counts as 0% inhibition H12E1 antibody treated samples exhibited ≥ 90% inhibition in parasite growth (Fig. 3c).

### mAb concentration dependence of parasite growth inhibition

**Fig. 4:**
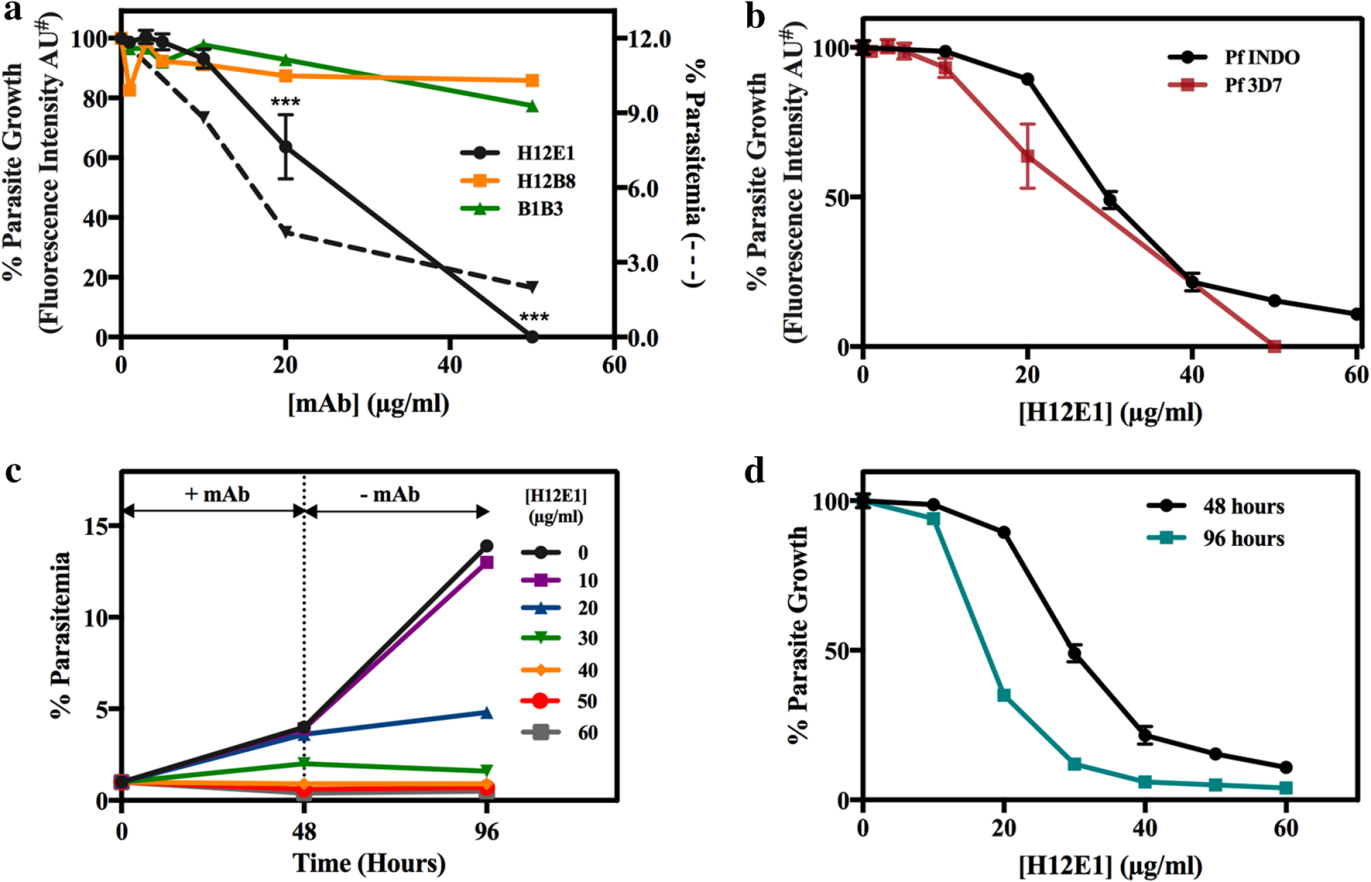
Growth inhibitory activity (GIA) of various mAbs directed against rPfeno. **a** Effect of varying concentrations of H12E1, H12B8 and B1B3 mAbs on growth of *P. falciparum* 3D7. **b** Effect of varying concentrations of H12E1 IgG on *P. falciparum* 3D7 (chloroquine sensitive) and *P. falciparum* INDO (chloroquine resistant) strains. **c** Effect of exposure of the parasite cultures to H12E1 antibody on subsequent growth. Two sets of tightly synchronized ring stage parasites (~ 1%) were cultured in presence of varying amounts (0–60 µg/ml) of H12E1 IgG. At 48 h, parasitemia was measured for the first set of cultures while the second set of cultures was washed free of H12E1 antibody and supplemented with fresh medium. This was grown for another 48 h (in absence of mAb) and % parasitemia was determined at 96 h. Results are presented as % parasitemia at 0, 48 and 96 h for different concentrations of H12E1 and (**d**) % parasitemia vs [H12E1]. Values are presented as mean ± SD for triplicate samples. AU^#^, arbitrary units; * Marks the samples that had significant growth inhibition as compared to the control samples (*p < 0.05, **p < 0.01, ***p < 0.001)

For independent verification of the growth neutralizing properties of H12E1, growth inhibition assays were also performed in Dr. D. Sahal’s laboratory at ICGEB, New Delhi, India. The dose dependence of growth inhibitory activity of H12E1 antibody along with two others (B1B3 and H12B8) was evaluated. Tightly synchronized ring stage parasites were treated with varying concentrations of purified mAbs (0–60 μg/ml). H12E1 mAb had a potent growth neutralizing effect with almost complete growth inhibition at ~ 50–60 µg/ml with EC_50_ ~ 21.3 ± 2.1 µg/ml. In a parallel measurement, effect of H12E1 was also determined by measuring % parasitemia microscopically (— line trace in Fig. 4a). EC_50_ value obtained from microscopy data was ~ 13 µg/ml (Table 1). Two other mAbs (B1B3 and H12B8) did not show any significant decline in parasite growth.

**Table 1:** Effective concentration of mAb H12E1 for 50% growth inhibition (EC_50_) values for *Plasmodium falciparum* growth in in vitro cultures

S No.	*P. falciparum* strain	EC_50_ (µg/ml)	Method
1	3D7	13.0	Microscopy data Fig. 4a
2	3D7	21.3 ± 2.1	SYBR dye data Fig. 4a
3	INDO	28.5 ± 2.9	SYBR dye data Fig. 4b
5	3D7	28.5 ± 2.7	48 h data Fig. 4d
6	3D7	16.6 ± 1.3	96 h data Fig. 4d

### H12E1 is a strain-transcending, growth-neutralizing antibody

For determining whether the growth inhibitory effect of H12E1 was strain specific, two different strains of *P. falciparum* were tested, namely 3D7 (chloroquine-sensitive strain) and INDO (chloroquine-resistant strain) (Fig. 4b). Both strains of *P. falciparum* showed very similar growth inhibition profiles. The estimated EC_50_ value for *P. falciparum* INDO was 28.5 ± 2.7 µg/ml. Although there are limited data on parasite strains, nonetheless, they are indicative of strain transcending nature of H12E1 induced growth inhibition.

### Inhibition of parasite growth in in vitro cultures is irreversible

The nature of antibody induced growth inhibitory effect on parasite (reversible or irreversible) was investigated in in vitro cultures. Culture samples containing synchronized *P. falciparum* infected RBCs (~ 1% pRBCs) were allowed to grow in the presence of several different concentrations of H12E1 (0–60 µg/ml). A replicate of the first set of cultures was also included in the experiment. After 48 h when a fresh cycle of merozoite invasion of RBCs had occurred, the first set of culture samples was used to determine the parasitemia. For assessing the effect of 48 h exposure of the parasite to H12E1 antibody on subsequent growth cycle, the antibody was removed from the second set of culture samples by washing with fresh RPMI culture medium and allowed to grow for an additional 48 h. Thus, in this set of cultures, pRBCs had an exposure to the antibody in the first 48 h while the next cycle of multiplication was in a mAb free environment. Growth of the parasite in presence of several different concentrations of H12E1 at 48 and 96 h was monitored by dye fluorescence assay. Percentage parasitemia in control sample ([H12E1] = 0 µg/ml) was also measured microscopically at all the three time points (0, 48 and 96 h). Parasite growth with time at various concentrations of the antibody is shown in Fig. 4c. In control samples (antibody free, [H12E1] = 0 µg/ml) parasitemia counts rose from the initial (0 h) ~ 1 to ~ 4%. In the second cycle (96 h) of growth, control parasitemia rose further from ~ 4 to 14%. Control samples had ~ 3.5-fold more parasites at 96 h compared to the 48-h count (Fig. 4c). In H12E1-treated samples at low levels of mAb (10 µg/ml), cultures had near normal growth (~ 13% parasitemia). At 20 and 30 µg/ml H12E1 (intermediate concentration range), % parasitemia increased to ~ 3 and 2%, respectively, after the first cycle of growth (at 48 h). These samples had ~ 25–50% inhibition of growth as compared to control. Interestingly, in the second cycle of multiplication (after washing away the antibody), there was barely any growth in culture samples treated with 20–30 µg/ml of H12E1. Percentage parasitemia increased from 3 to 4% in the 20 µg/ml antibody-treated sample while at 30 µg/ml there was a decrease (from ~ 2 to 0.9% parasitemia at 48 and 96 h, respectively). It was expected that % parasitemia would rise from ~ 3 to ~ 10% in samples treated with 20 µg/ml antibody and from 2 to ~ 7% in 30 µg/ml treated samples (3.5-fold increase similar to controls). The inability of the antibody exposed parasite from the first cycle to revive in the antibody free milieu in the second cycle indicates that the H12E1 antibody is ‘parasiticidal’ in action. Growth of the parasites in culture at various concentrations of H12E1 is shown in Fig. 4d. The EC_50_ values obtained from these titrations were 28.5 ± 2.7 (from 48 h data) and 16.6 ± 1.3 µg/ml (96 h data), respectively (Table 1). EC_50_ values estimated by different methods of parasitemia measurements (dye assay and microscopy) for two strains of *P. falciparum* ranged between 13.0 and 28.5 µg/ml (Table 1).

### Passive immunization of mice with H12E1 mAb protects against heterologous parasite challenge in vivo

**Fig. 5:**
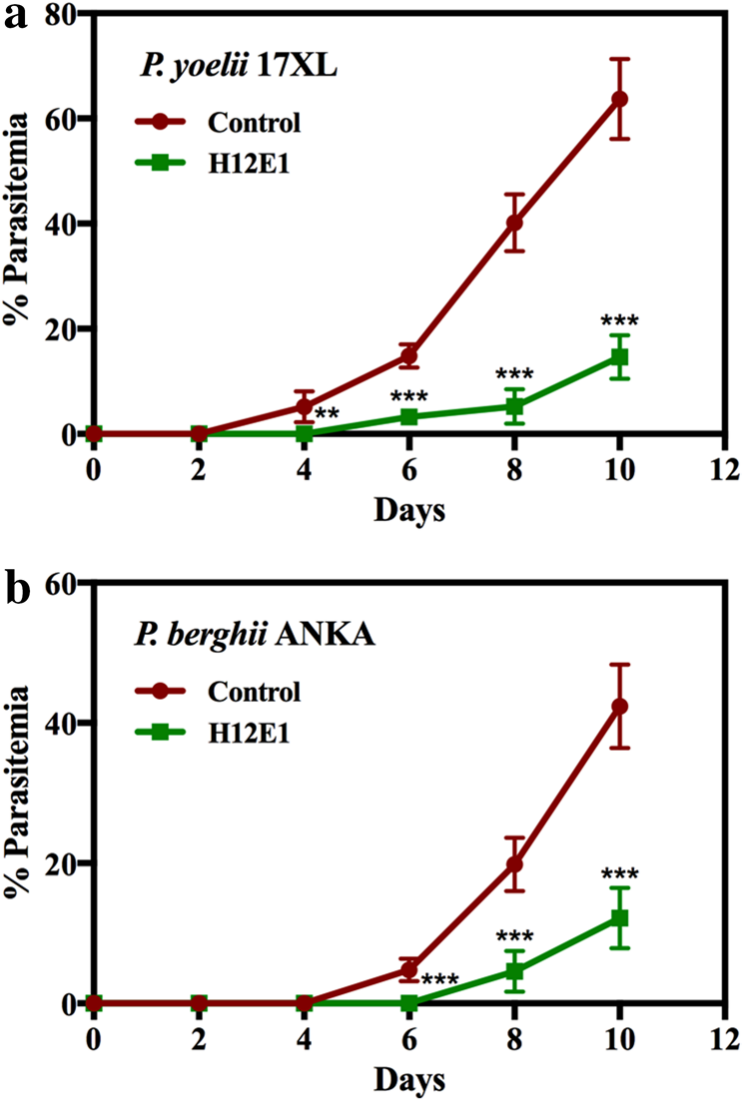
Effect of passive immunization with H12E1 mAb on parasitemia in *Plasmodium yoelii* 17XL (or *Plasmodium berghei* ANKA) infected mice. **a** Mice (n = 5) were administered with 0.75 mg/100 µl H12E1 (experimental) or pre-immune IgGs intravenously. All mice were challenged with ~ 1 × 10^5^ *P. yoelii* 17 XL pRBCs. Mean % parasitemia for the two groups of animals is shown with post challenge days. **b** Two groups of mice (n = 5 animals) were administered with 1 mg/100 µl H12E1 (experimental) or pre-immune IgGs (control). All mice were challenged with ~ 10^5^ *P. berghei* ANKA pRBCs. Passive transfer of H12E1 in infected mice resulted in significant reduction in rise of parasitemia in experimental group as compared to control group (**p < 0.01; ***p < 0.001)

Passive immunization experiments were performed in mice to assess the in vivo growth neutralizing ability of H12E1. In two different experiments, mice were challenged with *P. yoelii* 17XL or *P. berghei* strain ANKA and injected with purified IgG on the same day. The protective effect of H12E1 was assessed by measuring the effect of injected antibody on the parasitemia and comparing that with the pre-immune IgGs injected controls. Results obtained for two different species of mouse malarial parasites are shown in Fig. 5. In *P. yoelii* challenged group, passive infusion of H12E1 clearly attenuated parasitemia in experimental animals compared to the controls (Fig. 5a). The rise in parasitemia was delayed and remained low (~ 15%) in H12E1 injected mice for the first 10 days, while the parasitemia in the control mice shot up to ≥ 60%.

Similar results were obtained in the experiment where *P. berghei* strain ANKA was used as the infective agent (Fig. 5b). In this case also % parasitemia rose rapidly in controls reaching to > 50% by 10th day while in the experimental group, it was curtailed to < 15%. Statistical comparison of the experimental group with the control showed significant reduction in % parasitemia. For the duration that mAb remained in the system, parasitemia too remained under control. These experiments provided an effective way to test the in vivo protective potential of the antibody for parasite neutralization as have been done earlier [17, 41].

### Kinetic and thermodynamic measurements on interaction of rPfeno with mAb H12E1

**Fig. 6:**
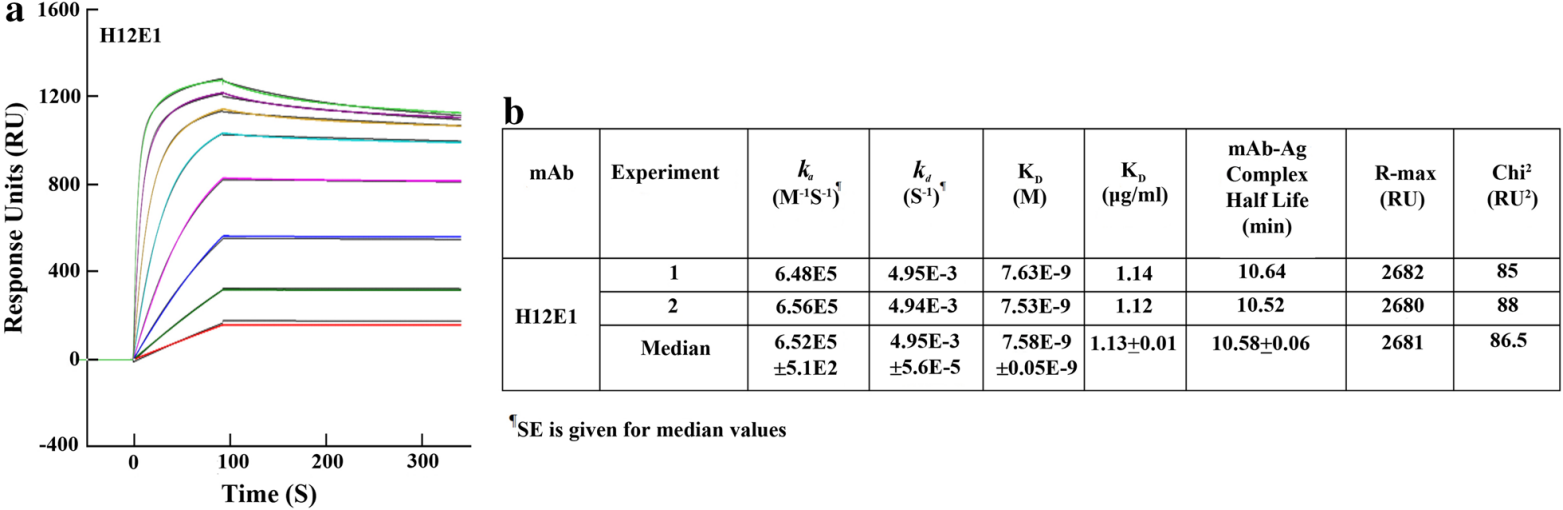
Determination of association-dissociation rate constants for interaction between rPfeno and mAb H12E1. Thermodynamic and kinetic parameters for rPfeno-H12E1 interaction were measured using Surface Plasmon Resonance. **a** Sensogram shown represents 1:1 binding of bivalent mAb with dimeric enolase. Measurements were made in duplicates with different traces representing various concentrations of mAb (0.78–100 nM) used with fixed concentration of rPfeno on the CM5 chip. **b** Best fit kinetic parameters were derived from the sensogram. Dissociation constant K_D_ was computed from association and dissociation rate constants. The half-life of mAb-Antigen complex was computed using t_1/2_ = (ln [0.5]/60*k_*d*_). Goodness of fit as judged by Chi^2^ was < 10% of R_max_

Binding affinity and half-life of an antibody-antigen complex are important determinants of an antibody’s potency to neutralize a pathogen [42, 43]. Direct interaction of H12E1 with rPfeno was monitored using Surface Plasmon Resonance (SPR). Binding sensograms obtained for H12E1 interaction with rPfeno are shown in Fig. 6a. These sensograms were analyzed by fitting the data to a 1:1 binding site model. The association rate constant for antibody-antigen interaction *k*_*a*_ = 6.5 × 10^5^/M/S. The best-fit value obtained by fitting each of the dissociation traces in the sensogram gave the dissociation rate constant *k*_*d*_= 4.95 × 10^−3^/S (Fig. 6b). The ratio of the two rate constants (*k*_*d*_*/k*_*a*_) yielded a dissociation constant K_D_ = 7.58 × 10^−9^ M. Considering the K_D_ value obtained here, it can be argued that at a mAb concentration of ~ 1–2 µg/ml, ~ 50% of the antigen would be in bound state. The value of the association rate constant obtained here falls well within the range suggested earlier (1 × 10^5^/M/S) [44]. A similar value was reported for PfRH5 (~ 2x10^6^/M/S) [43] for the kinetics of merozoite neutralization by antibodies. Using the dissociation rate constant (*k*_*d*_) for mAb-rPfeno complex, the half-life (t_1/2_ = ln [0.5]/60*k_*d*_) of rPfeno-H12E1 complex was also computed (t_1/2_ = 10.5 min). Since merozoites reinvade fresh erythrocytes in a couple of minutes of their egress, it requires high concentrations of high specificity antibodies that can bind rapidly (high *k*_*a*_) and stably (long t_1/2_) so as to have a substantial fraction of merozoites saturated with antibody to generate the inhibitory effect [44, 45]. The values obtained here for H12E1 interaction are well within the range assuming that the cell surface enolase has a binding behavior with H12E1 similar to free Pfeno in solution.

### Binding of EWGWS specific mAb to rPfeno did not affect enzyme activity

**Fig. 7:**
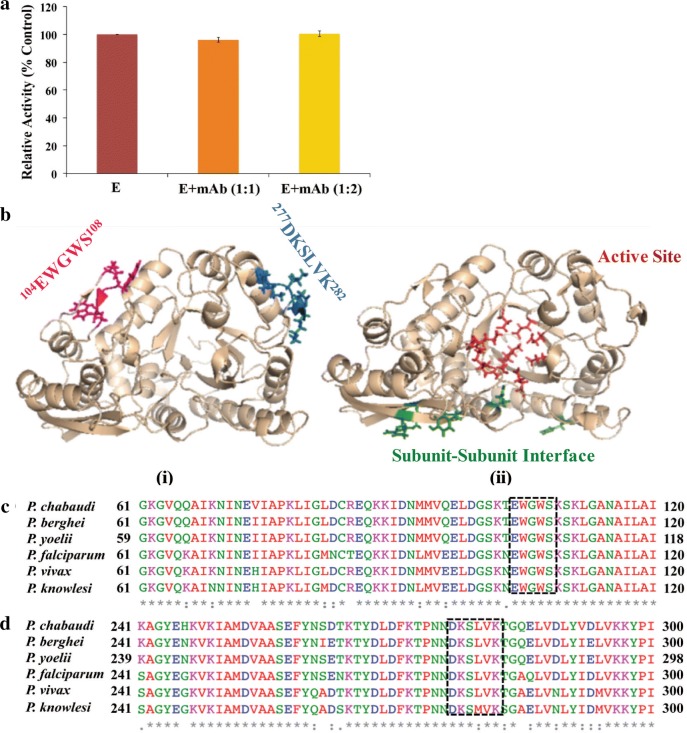
**a** Effect of mAb (H12E1) binding on the enzyme activity of rPfeno. Activity of enolase was assayed using 80 nmoles (2 µg) of rPfeno. For the measurement of antibody effect on enzyme activity, different amounts (80 and 160 nmoles) of H12E1 were mixed with rPfeno (80 nmoles) and incubated for 30 min before using for enolase activity assays. **b** 3D-structure of Pfeno was modelled using *Toxoplasma gondii* enolase 1 crystal structure as template (TgENO1; PDB: 3OTR). Residues that constitute the different functional regions are highlighted in different colours: plasminogen binding site (blue); active site residues (red); subunit–subunit interface (green); and, EWGWS (pink)—target epitope for growth neutralizing antibody. Note the non-overlapping nature of all four regions. **c**, **d** Homology comparison of enolase sequence in the vicinity of EWGWS and DKSLVK (plasminogen binding site) in various species of *Plasmodium*. Note the conserved sequence and their location in the protein

Enolase is a glycolytic enzyme that catalyzes the inter conversion of 2-phosphoglycerate and phosphoenolpyruvate. As *Plasmodium* has a single gene for enolase, inhibition of this activity could result in parasite growth inhibition. To determine whether the binding of H12E1 antibody had any effect on the enzyme activity, rPfeno (80 nmoles) was incubated with different amounts of mAb (0, 80 and 160 nMoles) and the activity of rPfeno-mAb complex was measured. The activity measurements showed that the binding of enolase to H12E1 had no effect on its catalytic activity (Fig. 7a) indicating that the antibody-binding motif in enolase is likely to be exclusive of the active site of the protein. The relative location of various functional domains in the molecule were examined using molecular modelling and visualisation tools. For this, a 3D-structure of Pfeno was modelled based on *Toxoplasma gondii* enolase 1(TgEno1) (PDB: 3OTR). Structural mapping of the four functional sites: (i) the plasminogen binding site ^277^DKSLVK^282^ (blue) [19]; (ii) the active site (residues - Glu175, Glu218, Asp253, Glu304, Asp331, Lys356, Arg385, Lys407 and His384) (red) [22]; (iii) the subunit–subunit interface region (residues—His198, Tyr59, Glu23, Arg416, Arg11 and Glu427) [46] (green); and, (iv) the EWGWS epitope (pink) (target for the growth inhibitory antibodies) showed them all to be non-overlapping (Fig. 7b). This was in line with the above observation that the binding of H12E1 to Pfeno did not affect the catalytic activity. Thus, the possibility of inhibition of glycolysis being the cause of antibody induced growth inhibition was excluded. Evidence presented here supports the conclusion that the potent growth inhibition arose solely due to the blockade of the merozoite surface enolase. The first three sites (i to iii) are conserved across all enolases and blockade of anyone of them in Pfeno will also block host enolases resulting in adverse physiological effects in the host system. Since EWGWS is present only in the parasite (apicomplexans) enolases, targeting this region could yield parasite specific growth inhibitory effects.

## Discussion

Enolase is a glycolytic enzyme with a highly conserved structure and multiple moonlighting functions [47, 48]. Several pathogens have enolase expressed on their cell surface where it assists in host tissue invasion [49–54]. In *Plasmodium*, enolase localizes in multiple organelles (diverse sub-cellular localization) where it is likely to participate in different physiological functions [18]. Presence of Pfeno on the surface of sporozoites, merozoites and ookinetes raised the possibility of its involvement in host cell invasion. Recently, anti-Pfeno antibodies have been shown to inhibit growth at erythrocytic and mosquito stages presumably by blocking the invasion by merozoites and ookinetes [19, 20, 23, 50]. Antibody induced inhibition of ookinete was mediated through the plasminogen receptor function of Pfeno, [19, 50] while the merozoite inhibition appeared to be mediated by blocking a unique epitope, EWGWS [23, 24]. Data presented here further validated that EWGWS is the target for the merozoite neutralizing antibodies. The inhibitory antibodies exert their effect on ookinete by blocking the binding of plasminogen to ^277^DKSLVK^282^ domain of Pfeno, while merozoite inhibitory antibody (H12E1) functions through the ^104^EWGWS^108^ epitope. The underlying molecular mechanisms in the two cases are likely to be quite different. In ookinetes, Pfeno functions as a receptor for plasminogen as well as a ligand for mosquito mid-gut epithelial cell receptor [19], whereas in merozoites, it is the EWGWS epitope of Pfeno that serves as a ligand for some yet to be identified receptor on human erythrocyte surface. The effect of anti-Pfeno antibodies on sporozoite invasion of hepatocytes has not yet been examined. It will be interesting to see whether anti-Pfeno antibodies can disrupt sporozoite invasion of hepatocytes.

The observed inhibition of growth shown by H12E1 mAb was ≥ 90% at < 100 µg/ml. There are very few reports where such potent inhibition of merozoite invasion in in vitro cultures has been reported. Some of the mAbs raised against *P. falciparum* reticulocyte binding protein homolog 5 (PfRH5) had shown similar inhibition [55]. H12E1 antibody was quite effective against multiple species of *Plasmodium* (*P. yoelii, P. berghei* and *P. falciparum*) and different strains of *P. falciparum* (3D7 and INDO). Given the highly conserved nature of the target sequence and its location in the polypeptide (Fig. 7c), it is not surprising that the antibody has species and strain transcending parasite neutralization activity. The ability of this antibody to block erythrocyte invasion with high efficiency and inhibit growth in heterologous species and strains other than *P. falciparum* 3D7 makes it a promising candidate for a blood stage anti malaria vaccine. One of the major limitations of this epitope is the poor immunogenicity of EWGWS. As all plant enolases have this epitope [22, 56], humans (or other vertebrate hosts) get exposed to this antigen early on in their life (through food). There have been suggestions that oral tolerance to food antigens could induce systemic suppression [57]. Such suppression could be one of the factors responsible for poor immunogenicity of EWGWS. Observation of partial protection in EWGWS immunized mice was largely due to low titers of induced antibodies [23]. Thus, for greater efficacy in protection against malaria, this epitope will need to be presented in a highly immunogenic context, employing novel methods of antigen presentation [55, 58–61].

Several antigens derived from the three stages of parasite life cycle (i.e. pre-erythrocytic, erythrocytic and mosquito stages) have been tested for their ability to protect against malaria and block its transmission [8, 9, 62]. Some of the erythrocytic stage antigens, mostly of merozoite origin, have undergone phase I/II level trials [12]. Apical membrane antigen 1 (AMA-1) that is expressed by both sporozoites and merozoites plays a key role in invasion of hepatocytes and erythrocytes, showed strain specific reduction in malaria cases, but offered no clinical protection [13, 63, 64]. Other vaccine candidates such as Merozoite Surface Protein 1 (MSP 1), Erythrocyte Binding Antigen Region II (EBA 175) etc. have also undergone Phase I and Phase II level trials [65–67]. In all these efforts, the antibody response failed to cover antigenic polymorphism and yielded only low efficacy allele specific protective response [12]. Multi-stage vaccines containing candidate antigens from multiple stages have also been tested [68, 69]. Extensive efforts to cover all genetic variations in field strains have not succeeded mostly due to the failure to overcome the issue of polymorphism. Stage specific expression, high degree of polymorphism and requirement for very high titers of induced antibodies has hindered any meaningful outcome from these efforts. Recently, PfRH5 has emerged as a leading antigen for erythrocytic stage vaccine [70]. PfRH5 is a merozoite adhesin essential for erythrocyte invasion and is susceptible to vaccine inducible strain transcending parasite neutralizing antibodies [71–73]. PfRH5 has critical non-redundant function during merozoite invasion of erythrocytes [74] and does not appear to be a target of antibodies acquired in natural immunity [72]. Results from a recent Phase Ia trial have been very promising [75]. There are some interesting parallels between Pfeno and PfRH5. Both antigens have a high degree of sequence conservation (none or low genetic polymorphism) [73, 76–78], both are targets for strain transcending parasite neutralizing antibodies, mAbs directed towards certain specific epitopes in these proteins have shown strong growth inhibition [55] and both appear to be under low level natural immune pressure. However, an added advantage with Pfeno is that it has cell surface expression at all the three invasive stages [18], two (merozoites and ookinetes) of which are known targets of parasite neutralizing antibodies [19, 20, 23]. Thus, it could be developed into a ‘two stage’ (erythrocytic and transmission blocking) vaccine candidate. The possibility of anti-Pfeno antibodies targeting sporozoites should also be explored.

For the development of a multivalent vaccine that is effective against multiple species and strains of the parasite, with an ability to neutralize growth at multiple stages, one will need to combine several antigens [15, 17, 62]. Epitope based approaches for each target antigens are also anticipated in near future. Hence, antigens like Pfeno with identified protective epitopes, ability to neutralize multiple species/strains and with potential to target multiple stages in parasite life cycle could be the promise of future.

## Conclusions

In this paper, we have described the generation of a monoclonal antibody H12E1 that has a potent merozoite neutralizing activity and targets the EWGWS epitope in Pfeno. The mAb induced an almost complete inhibition of parasite growth in in vitro cultures of the asexual stages (> 90% inhibition at < 100 µg/ml). Active immunization of mice with an antigenic peptide containing EWGWS epitope is known to confer partial protection [23]. Passive immunization of infected mice with H12E1 (anti-EWGWS antibody) led to drastic reduction in parasitemia, indicating EWGWS to be a valid target for parasite neutralizing antibodies. Limited data provided on growth inhibitory activity of the antibody on two different strains of *P. falciparum* (3D7 and INDO) and three different species of *Plasmodium* (*P. falciparum, P. yoelii* and *P. berghei*) indicates the species and strain transcending nature of this epitope. *Plasmodium* spp. enolase has thus emerged as a unique antigen that has surface expression at all three invasive stages; is an invasion target in at least two stages (merozoites and ookinetes); the target epitope of which is highly conserved, is monomorphic and has the ability to induce protective antibodies on immunization. Poor immunogenicity of this epitope is a concern that needs to be addressed. Thus, antigenic epitope EWGWS from Pfeno could be a good candidate for the development of a two-stage malaria vaccine.

## Additional files

**Additional file 1.** Purification, specificity and isotyping of mAbs. Three mAbs that had high reactivity with WT-ePfeno were purified. (A) Coomassie stained 10% SDS-PAGE for the Protein-A Sepharose purified IgGs from hybridoma supernatants of the three mAbs. Two bands corresponding to heavy and light chain of antibody are observed. (B) Western blot of purified rPfeno with each mAb and (C) isotyping of mAbs. All three mAbs were of IgG2b class of immunoglobulins.

**Additional file 2.** Quantitation of un-ruptured schizonts in anti-rPfeno antibody treated cultures at 48 h post antibody addition. Control samples had no schizonts. Data are expressed as percent of total Schizonts present at 24 h post antibody addition (see Fig. 3).

## Abbreviations

ABTS: 2,2′-azino-bis[3-ethylbenzothiazoline-6-sulfonic acid]-diammonium salt
Ab: antibody
Ag: antigen
CQ: chloroquine
pRBCs: parasitized red blood cells
EDC: *N*-(3-dimethylaminopropyl)-*N*′-ethylcarbodiimide hydrochloride
GIA: growth inhibitory activity
h: hours
mAb: monoclonal antibody
NHS: *N*-hydroxysuccinimide
PBS: phosphate buffer saline
Pfeno: *Plasmodium falciparum* enolase
SPR: Surface Plasmon Resonance
∆^5^-rPfeno: pentapeptide deletion variant of rPfeno
S108G-rPfeno: S108G variant of rPfeno
Rec-NP: recombinant nanoparticles
WT-rPfeno: wild type recombinant *Plasmodium falciparum* enolase
WT-NP: wild type nanoparticles
Yeno: yeast enolase
