# Supplementary material for: Strain-transcending neutralization of malaria parasite by antibodies against Plasmodium falciparum enolase
Source: Malar J. 2018 Aug 20;17:304. doi: 10.1186/s12936-018-2455-6 (PMC6102825; doi:10.1186/s12936-018-2455-6)
Supplement: Supplementary file 1 — Additional file 1. Purification, specificity and isotyping of mAbs. Three mAbs that had high reactivity with WT-ePfeno were purified. (A) Coomassie stained 10% SDS-PAGE for the Protein-A Sepharose purified IgGs from hybridoma supernatants of the three mAbs. Two bands corresponding to heavy and light chain of antibody are observed. (B) Western blot of purified rPfeno with each mAb and (C) isotyping of mAbs. All three mAbs were of IgG2b class of immunoglobulins. [file 12936_2018_2455_MOESM1_ESM.docx]

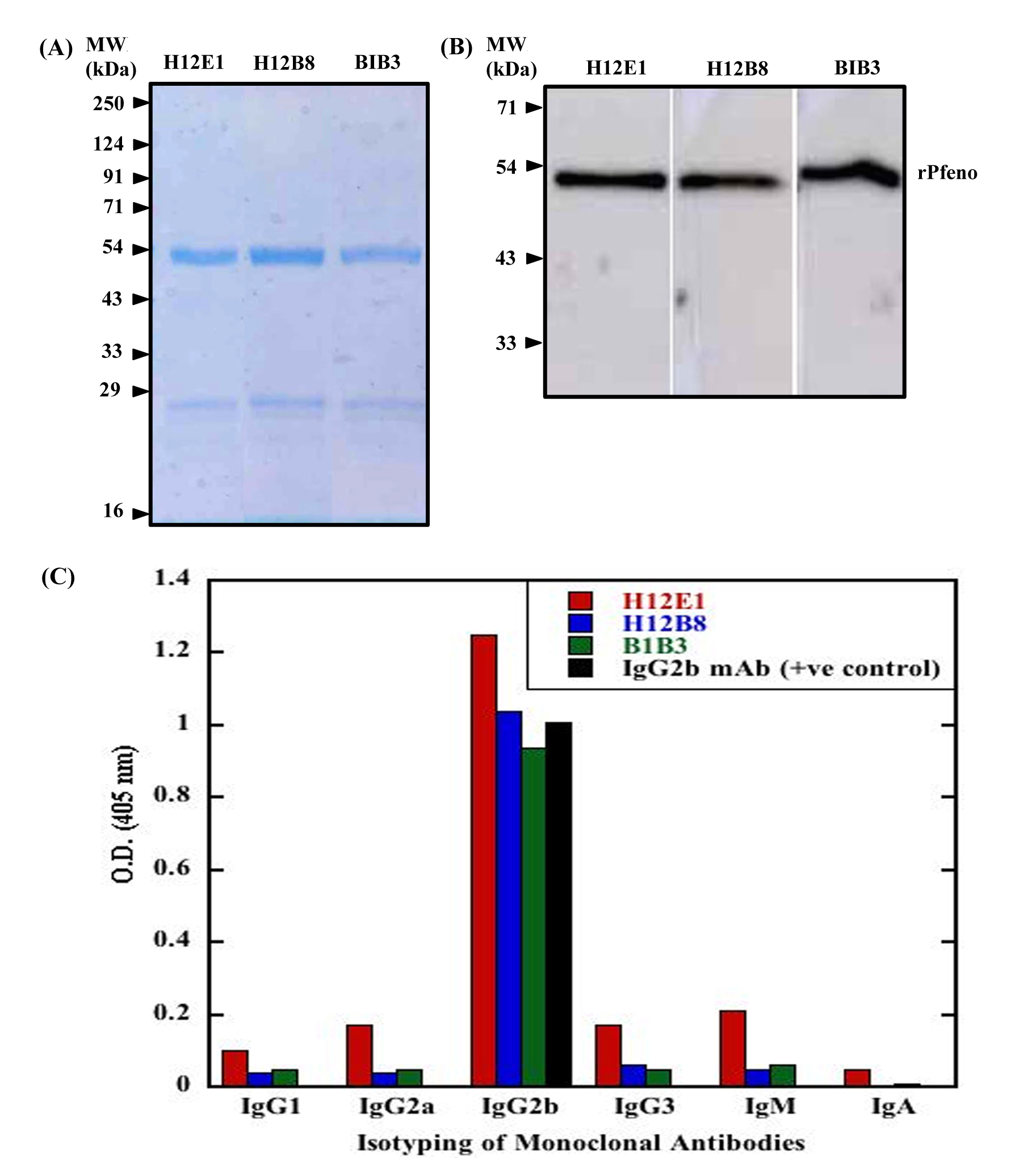


**Additional file 1:** **Purification, specificity and isotyping of mAbs.** Three mAbs that had high reactivity with WT-ePfeno were purified. (A) Coomassie stained 10% SDS-PAGE for the Protein-A Sepharose purified IgGs from hybridoma supernatants of the three mAbs. Two bands corresponding to heavy and light chain of antibody are observed. (B) Western blot of purified rPfeno with each mAb and (C) isotyping of mAbs. All three mAbs were of IgG2b class of immunoglobulins.
