# Supplementary material for: Strain-transcending neutralization of malaria parasite by antibodies against Plasmodium falciparum enolase
Source: Malar J. 2018 Aug 20;17:304. doi: 10.1186/s12936-018-2455-6 (PMC6102825; doi:10.1186/s12936-018-2455-6)
Supplement: Supplementary file 2 — Additional file 2. Quantitation of un-ruptured schizonts in anti-rPfeno antibody treated cultures at 48 h post antibody addition. Control samples had no schizonts. Data are expressed as percent of total Schizonts present at 24 h post antibody addition (see Fig. 3). [file 12936_2018_2455_MOESM2_ESM.docx]

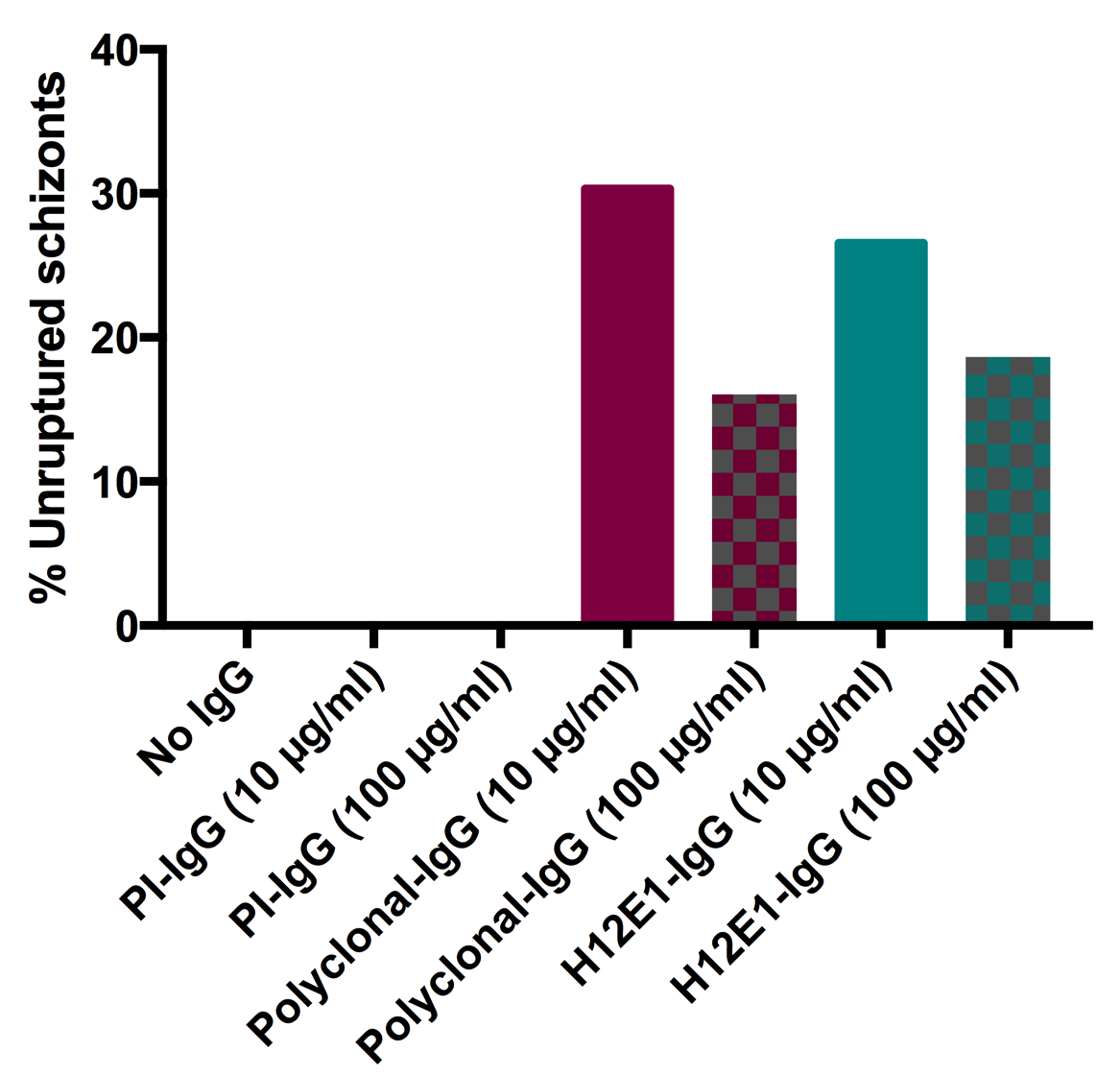


Additional file 2: Quantitation of un-ruptured schizonts in anti-rPfeno antibody treated cultures at 48 hrs post antibody addition. Control samples had no schizonts. Data are expressed as percent of total Schizonts present at 24 hours post antibody addition (see Figure 3).
